# 1,2-Dimethyl-1,4-diazo­niabicyclo­[2.2.2]octane tetra­chloridocuprate(II)

**DOI:** 10.1107/S1600536811025347

**Published:** 2011-07-02

**Authors:** Tao Rong

**Affiliations:** a Ordered Matter Science Research Center, Southeast University, Nanjing 210096, People’s Republic of China

## Abstract

In the title compound, (C_8_H_18_N_2_)[CuCl_4_], torsion angles on the ethyl­ene bridges of the 1,4-diazo­niabicyclo­[2.2.2]octane fragment are in the range 11.9 (5)–15.0 (5)° and the [CuCl_4_]^2−^ anion has a strongly distorted tetra­hedral geometry. The cation is connected to the anion *via* three-center N—H⋯Cl hydrogen bonds.

## Related literature

For similar compounds exhibiting phase transition, see: Corzo-Suárez *et al.* (1997 ▶); Katrusiak (2000 ▶); Sun & Jin (2002 ▶).
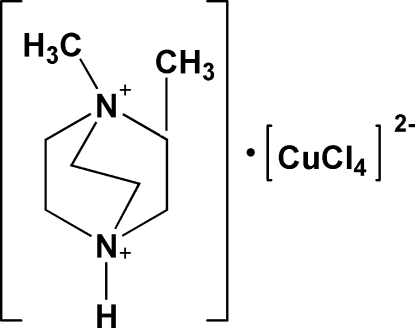


## Experimental

### 

#### Crystal data

(C_8_H_18_N_2_)[CuCl_4_]
*M* *_r_* = 347.58
Orthorhombic, 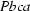

*a* = 13.347 (3) Å
*b* = 14.187 (3) Å
*c* = 14.408 (3) Å
*V* = 2728.2 (9) Å^3^
*Z* = 8
Mo *K*α radiation
μ = 2.36 mm^−1^
*T* = 293 K
0.20 × 0.20 × 0.20 mm

#### Data collection

Rigaku SCXmini diffractometer
Absorption correction: multi-scan (*CrystalClear*; Rigaku, 2005 ▶) *T*_min_ = 0.618, *T*_max_ = 0.624
26550 measured reflections
3131 independent reflections
2455 reflections with *I* > 2σ(*I*)
*R*_int_ = 0.059

#### Refinement

*R*[*F* ^2^ > 2σ(*F* ^2^)] = 0.050
*wR*(*F* ^2^) = 0.123
*S* = 1.12
3131 reflections
136 parameters
H-atom parameters constrained
Δρ_max_ = 0.60 e Å^−3^
Δρ_min_ = −0.46 e Å^−3^

### 

Data collection: *CrystalClear* (Rigaku, 2005 ▶); cell refinement: *CrystalClear*; data reduction: *CrystalClear*; program(s) used to solve structure: *SHELXS97* (Sheldrick, 2008 ▶); program(s) used to refine structure: *SHELXL97* (Sheldrick, 2008 ▶); molecular graphics: *DIAMOND* (Brandenburg & Putz, 2005 ▶); software used to prepare material for publication: *SHELXL97*.

## Supplementary Material

Crystal structure: contains datablock(s) I, global. DOI: 10.1107/S1600536811025347/gk2387sup1.cif

Structure factors: contains datablock(s) I. DOI: 10.1107/S1600536811025347/gk2387Isup2.hkl

Additional supplementary materials:  crystallographic information; 3D view; checkCIF report

## Figures and Tables

**Table 1:** Hydrogen-bond geometry (Å, °)

*D*—H⋯*A*	*D*—H	H⋯*A*	*D*⋯*A*	*D*—H⋯*A*
N1—H1⋯Cl1	0.91	2.54	3.271 (4)	138
N1—H1⋯Cl3	0.91	2.57	3.229 (4)	130

**Fig. 1.**
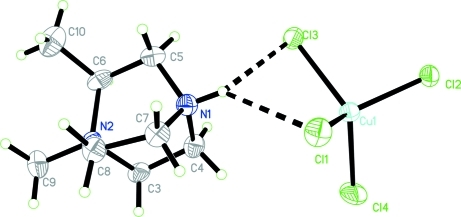
The molecular structure of the title compound, with the atomic numbering scheme. Displacement ellipsoids are drawn at the 30% probability level. Dashed lines indicate hydrogen bonds.

**Fig. 2.**
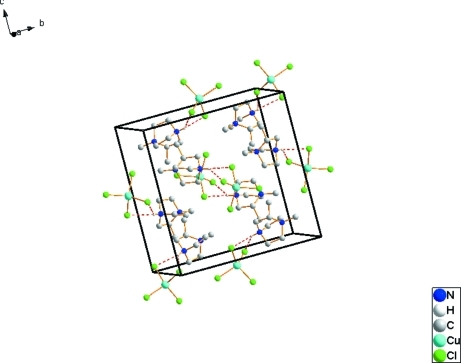
Crystal packing of the title compound viewed along the b axis. Dashed lines indicate hydrogen bonds.

## supplementary crystallographic information

### Comment

The synthesis and characterization of copper halides in which organic ligands link metal centres have attracted much attention. One of the reasons for that is the fact that the copper environment of these compounds can adopt different geometries: tetrahedral, pseudo-tetrahedral or square-planar (Corzo-Suárez *et al.*, 1997). This coordination variety allows the study of the relationship between the different geometries and the structural and magnetic properties of these compounds.

The title compound at room temperature crystallizes in the centrosymmetric Pbca space group and is paraelectric. It contains an isolated distorted (compressed) tetrahedral [CuCl_4_]^2-^ anion, and a protonated (C_8_H_18_N_2_)^2+^ cation. In this salt, the N—H^+^ group of the dication forms bifurcated hydrogen bond to two chloride ligands of the adjacent [CuCl_4_]^2-^ anion.

In the cation the C9—N2—C6—C10 torsion angle is 47.9 (6)° and the DABCO fragment is distorted as indicated by the N1—C—C—N2 torsion angles, which range from 11.9 (5)-15.0 (5)° . In contrast to the present case, disorder of the DABCO unit is frequently observed in DABCO salts, such as in DABCO–perchloric acid (1:1) (Katrusiak, 2000) and DABCO–maleic acid (1:2) (Sun & Jin, 2002).

In [CuCl_4_]^2-^ anion distortion from tetrahedral geometry is typically measured by the value of the *trans* Cl—Cu—Cl angle and by the dihedral angle between CuCl_2_ planes. In the present case, the two '*trans'* angles are 131.30°(5) and 131.22° (6) and the dihedral angle between the CuCl_2_ planes is 65.72 (5).

The dielectric measurements (capacitance and dielectric loss measurements) on the powder samples pressed into tablets with a conducting carbon glue depositing on it, were carried out with an automatic impedance TongHui2828 Analyzer. Dielectric permittivity of the compound was tested to investigate the possibility of ferroelectric phase transitions. In the temperature range 80- 423 K no dielectric anomaly was observed revealing no phase transition in the studied temperature range.

### Experimental

To a mixture of iodomethane (10 mmol) and chloroform (15 ml) at 273 K was added dropwise a chloroform solution of 2-methyl-1,4-diazabicyclo[2.2.2]octane (10.2 mmol) resulting in 1,2-methyl-1,4-diazabicyclo[2.2.2]octane iodide. To the mixture of 1,2-methyl-1,4-diazabicyclo[2.2.2]octane iodide (4 mmol,1.07 g) and water (7 ml), concentrated hydrochloric acid (12 mmol) was added dropwise. Concentrated hydrochloric acid was also added dropwise to a mixture of CuCl_2_ (2 mmol,0.341 g) and ethanol (5 ml). The two solutions were then mixed and stirred for 20 minutes. The resulting precipitate was dissolved in water. Yellow crystals suitable for X-ray analysis were formed after several weeks on slow evaporation of the solvent at room temperature (m.p. > 473 K).

### Refinement

All the H atoms were positioned geometrically (C-H = 0.96-0.97 Å; N-H = 0.91 Å) and in the refinement process were allowed to ride on their carrier atoms with *U*_iso_(H) = 1.2*U*_eq_(C, N).

### Crystal data

**Table:**

(C_8_H_18_N_2_)[CuCl_4_]	*F*(000) = 1416
*M**_r_* = 347.58	*D*_x_ = 1.692 Mg m^−^^3^
Orthorhombic, *P**b**c**a*	Mo *K*α radiation, λ = 0.71073 Å
Hall symbol: -P 2ac 2ab	Cell parameters from 7516 reflections
*a* = 13.347 (3) Å	θ = 3.1–27.5°
*b* = 14.187 (3) Å	µ = 2.36 mm^−^^1^
*c* = 14.408 (3) Å	*T* = 293 K
*V* = 2728.2 (9) Å^3^	Prism, yellow
*Z* = 8	0.20 × 0.20 × 0.20 mm

### Data collection

**Table:**

Rigaku SCXmini diffractometer	3131 independent reflections
Radiation source: fine-focus sealed tube	2455 reflections with *I* > 2σ(*I*)
graphite	*R*_int_ = 0.059
Detector resolution: 13.6612 pixels mm^-1^	θ_max_ = 27.5°, θ_min_ = 3.1°
φ scan	*h* = −17→17
Absorption correction: multi-scan (*CrystalClear*; Rigaku, 2005)	*k* = −18→18
*T*_min_ = 0.618, *T*_max_ = 0.624	*l* = −18→18
26550 measured reflections	

### Refinement

**Table:**

Refinement on *F*^2^	Primary atom site location: structure-invariant direct methods
Least-squares matrix: full	Secondary atom site location: difference Fourier map
*R*[*F*^2^ > 2σ(*F*^2^)] = 0.050	Hydrogen site location: inferred from neighbouring sites
*wR*(*F*^2^) = 0.123	H-atom parameters constrained
*S* = 1.12	*w* = 1/[σ^2^(*F*_o_^2^) + (0.0447*P*)^2^ + 4.3147*P*] where *P* = (*F*_o_^2^ + 2*F*_c_^2^)/3
3131 reflections	(Δ/σ)_max_ < 0.001
136 parameters	Δρ_max_ = 0.60 e Å^−^^3^
0 restraints	Δρ_min_ = −0.46 e Å^−^^3^

### Special details

**Table:**

Geometry. All e.s.d.'s (except the e.s.d. in the dihedral angle between two l.s. planes) are estimated using the full covariance matrix. The cell e.s.d.'s are taken into account individually in the estimation of e.s.d.'s in distances, angles and torsion angles; correlations between e.s.d.'s in cell parameters are only used when they are defined by crystal symmetry. An approximate (isotropic) treatment of cell e.s.d.'s is used for estimating e.s.d.'s involving l.s. planes.
Refinement. Refinement of *F*^2^ against ALL reflections. The weighted *R*-factor *wR* and goodness of fit *S* are based on *F*^2^, conventional *R*-factors *R* are based on *F*, with *F* set to zero for negative *F*^2^. The threshold expression of *F*^2^ > σ(*F*^2^) is used only for calculating *R*-factors(gt) *etc*. and is not relevant to the choice of reflections for refinement. *R*-factors based on *F*^2^ are statistically about twice as large as those based on *F*, and *R*- factors based on ALL data will be even larger.

### Fractional atomic coordinates and isotropic or equivalent isotropic displacement parameters (Å^2^)

**Table:**

	*x*	*y*	*z*	*U*_iso_*/*U*_eq_	
N2	0.0533 (2)	0.2734 (2)	0.3412 (2)	0.0377 (7)	
N1	0.1522 (3)	0.1255 (2)	0.3722 (2)	0.0435 (8)	
H1	0.1883	0.0724	0.3840	0.052*	
C4	0.0924 (3)	0.1493 (3)	0.4561 (3)	0.0478 (10)	
H4A	0.1354	0.1508	0.5103	0.057*	
H4B	0.0411	0.1020	0.4660	0.057*	
C7	0.2224 (3)	0.2034 (3)	0.3505 (3)	0.0471 (10)	
H7A	0.2677	0.1844	0.3013	0.057*	
H7B	0.2619	0.2189	0.4049	0.057*	
C3	0.0442 (3)	0.2452 (3)	0.4416 (2)	0.0455 (10)	
H3A	−0.0259	0.2426	0.4591	0.055*	
H3B	0.0771	0.2917	0.4804	0.055*	
C8	0.1625 (3)	0.2886 (3)	0.3202 (3)	0.0463 (10)	
H8A	0.1863	0.3442	0.3526	0.056*	
H8B	0.1713	0.2987	0.2541	0.056*	
C9	−0.0041 (4)	0.3621 (3)	0.3257 (4)	0.0610 (13)	
H9A	0.0240	0.4116	0.3629	0.091*	
H9B	−0.0729	0.3526	0.3429	0.091*	
H9C	−0.0005	0.3793	0.2613	0.091*	
C5	0.0852 (4)	0.1073 (3)	0.2931 (3)	0.0571 (12)	
H5A	0.0477	0.0496	0.3034	0.069*	
H5B	0.1241	0.1001	0.2367	0.069*	
C6	0.0129 (3)	0.1911 (3)	0.2836 (3)	0.0511 (11)	
H6A	−0.0509	0.1720	0.3115	0.061*	
C10	−0.0061 (5)	0.2090 (5)	0.1848 (4)	0.0870 (18)	
H10D	−0.0310	0.1526	0.1561	0.130*	
H10A	0.0552	0.2276	0.1551	0.130*	
H10B	−0.0547	0.2584	0.1786	0.130*	
Cu1	0.22240 (4)	−0.07018 (4)	0.54958 (3)	0.04302 (17)	
Cl2	0.23258 (8)	−0.22010 (7)	0.59863 (7)	0.0472 (3)	
Cl3	0.12745 (8)	−0.09541 (8)	0.42108 (7)	0.0464 (3)	
Cl1	0.33647 (9)	0.02455 (9)	0.48415 (9)	0.0607 (3)	
Cl4	0.18698 (12)	−0.00003 (10)	0.68313 (9)	0.0759 (4)	

### Atomic displacement parameters (Å^2^)

**Table:**

	*U*^11^	*U*^22^	*U*^33^	*U*^12^	*U*^13^	*U*^23^
N2	0.0365 (16)	0.0442 (18)	0.0324 (16)	−0.0054 (14)	0.0005 (13)	0.0043 (14)
N1	0.0467 (19)	0.0416 (18)	0.0421 (19)	−0.0007 (15)	0.0007 (16)	0.0007 (15)
C4	0.051 (2)	0.055 (3)	0.037 (2)	−0.002 (2)	0.0083 (19)	0.0096 (19)
C7	0.037 (2)	0.055 (2)	0.049 (2)	−0.0055 (19)	0.0082 (18)	0.004 (2)
C3	0.055 (2)	0.054 (2)	0.0273 (19)	−0.003 (2)	0.0094 (18)	−0.0013 (17)
C8	0.039 (2)	0.052 (2)	0.048 (2)	−0.0090 (19)	0.0066 (19)	0.007 (2)
C9	0.062 (3)	0.054 (3)	0.067 (3)	0.010 (2)	0.002 (2)	0.013 (2)
C5	0.070 (3)	0.053 (3)	0.048 (3)	−0.003 (2)	−0.010 (2)	−0.011 (2)
C6	0.043 (2)	0.071 (3)	0.040 (2)	−0.013 (2)	−0.0079 (19)	−0.007 (2)
C10	0.086 (4)	0.106 (5)	0.069 (4)	0.007 (4)	−0.016 (3)	0.011 (3)
Cu1	0.0504 (3)	0.0431 (3)	0.0355 (3)	0.0047 (2)	0.0013 (2)	−0.0021 (2)
Cl2	0.0558 (6)	0.0447 (6)	0.0411 (5)	0.0091 (5)	0.0000 (5)	0.0026 (4)
Cl3	0.0483 (6)	0.0482 (6)	0.0428 (5)	0.0009 (5)	−0.0049 (4)	0.0037 (4)
Cl1	0.0493 (6)	0.0646 (8)	0.0682 (8)	−0.0106 (5)	−0.0021 (6)	0.0065 (6)
Cl4	0.1097 (11)	0.0628 (8)	0.0550 (8)	0.0211 (8)	0.0109 (7)	−0.0202 (6)

### Geometric parameters (Å, °)

**Table:**

N2—C9	1.491 (5)	C8—H8B	0.9700
N2—C8	1.504 (5)	C9—H9A	0.9600
N2—C3	1.505 (5)	C9—H9B	0.9600
N2—C6	1.530 (5)	C9—H9C	0.9600
N1—C5	1.472 (5)	C5—C6	1.538 (7)
N1—C7	1.483 (5)	C5—H5A	0.9700
N1—C4	1.486 (5)	C5—H5B	0.9700
N1—H1	0.9100	C6—C10	1.469 (6)
C4—C3	1.520 (6)	C6—H6A	0.9800
C4—H4A	0.9700	C10—H10D	0.9600
C4—H4B	0.9700	C10—H10A	0.9600
C7—C8	1.513 (6)	C10—H10B	0.9600
C7—H7A	0.9700	Cu1—Cl4	2.2172 (13)
C7—H7B	0.9700	Cu1—Cl1	2.2390 (13)
C3—H3A	0.9700	Cu1—Cl2	2.2455 (12)
C3—H3B	0.9700	Cu1—Cl3	2.2719 (12)
C8—H8A	0.9700		
			
C9—N2—C8	110.3 (3)	N2—C8—H8B	109.7
C9—N2—C3	109.1 (3)	C7—C8—H8B	109.7
C8—N2—C3	108.0 (3)	H8A—C8—H8B	108.2
C9—N2—C6	112.5 (3)	N2—C9—H9A	109.5
C8—N2—C6	110.0 (3)	N2—C9—H9B	109.5
C3—N2—C6	106.9 (3)	H9A—C9—H9B	109.5
C5—N1—C7	110.5 (3)	N2—C9—H9C	109.5
C5—N1—C4	110.1 (3)	H9A—C9—H9C	109.5
C7—N1—C4	109.9 (3)	H9B—C9—H9C	109.5
C5—N1—H1	108.7	N1—C5—C6	108.3 (3)
C7—N1—H1	108.7	N1—C5—H5A	110.0
C4—N1—H1	108.7	C6—C5—H5A	110.0
N1—C4—C3	108.6 (3)	N1—C5—H5B	110.0
N1—C4—H4A	110.0	C6—C5—H5B	110.0
C3—C4—H4A	110.0	H5A—C5—H5B	108.4
N1—C4—H4B	110.0	C10—C6—N2	117.0 (4)
C3—C4—H4B	110.0	C10—C6—C5	109.1 (4)
H4A—C4—H4B	108.3	N2—C6—C5	108.7 (3)
N1—C7—C8	108.8 (3)	C10—C6—H6A	107.2
N1—C7—H7A	109.9	N2—C6—H6A	107.2
C8—C7—H7A	109.9	C5—C6—H6A	107.2
N1—C7—H7B	109.9	C6—C10—H10D	109.5
C8—C7—H7B	109.9	C6—C10—H10A	109.5
H7A—C7—H7B	108.3	H10D—C10—H10A	109.5
N2—C3—C4	109.6 (3)	C6—C10—H10B	109.5
N2—C3—H3A	109.8	H10D—C10—H10B	109.5
C4—C3—H3A	109.8	H10A—C10—H10B	109.5
N2—C3—H3B	109.8	Cl4—Cu1—Cl1	103.94 (6)
C4—C3—H3B	109.8	Cl4—Cu1—Cl2	99.49 (5)
H3A—C3—H3B	108.2	Cl1—Cu1—Cl2	131.30 (5)
N2—C8—C7	109.8 (3)	Cl4—Cu1—Cl3	131.22 (6)
N2—C8—H8A	109.7	Cl1—Cu1—Cl3	97.52 (5)
C7—C8—H8A	109.7	Cl2—Cu1—Cl3	98.11 (4)
			
C5—N1—C4—C3	−69.2 (4)	N1—C7—C8—N2	11.9 (5)
C7—N1—C4—C3	52.8 (4)	C7—N1—C5—C6	−69.6 (5)
C5—N1—C7—C8	54.3 (5)	C4—N1—C5—C6	52.0 (5)
C4—N1—C7—C8	−67.5 (4)	C9—N2—C6—C10	47.8 (6)
C9—N2—C3—C4	173.6 (4)	C8—N2—C6—C10	−75.6 (5)
C8—N2—C3—C4	−66.5 (4)	C3—N2—C6—C10	167.4 (4)
C6—N2—C3—C4	51.8 (4)	C9—N2—C6—C5	171.9 (4)
N1—C4—C3—N2	12.8 (5)	C8—N2—C6—C5	48.6 (4)
C9—N2—C8—C7	171.1 (4)	C3—N2—C6—C5	−68.5 (4)
C3—N2—C8—C7	51.9 (4)	N1—C5—C6—C10	143.7 (4)
C6—N2—C8—C7	−64.3 (4)	N1—C5—C6—N2	15.0 (5)

### Hydrogen-bond geometry (Å, °)

**Table:**

*D*—H···*A*	*D*—H	H···*A*	*D*···*A*	*D*—H···*A*
N1—H1···Cl1	0.91	2.54	3.271 (4)	138
N1—H1···Cl3	0.91	2.57	3.229 (4)	130
